# Surface Modification of Polytetrafluoroethylene and Polycaprolactone Promoting Cell-Selective Adhesion and Growth of Valvular Interstitial Cells

**DOI:** 10.3390/jfb13020070

**Published:** 2022-06-01

**Authors:** Matthias Gabriel, Christian Bollensdorff, Christophe Michel Raynaud

**Affiliations:** 1 Department of Prosthodontics, Geriatric Dentistry and Craniomandibular Disorders, Charité-Universitätsmedizin Berlin, Corporate Member of Freie Universität Berlin, Humboldt-Universität zu Berlin, and Berlin Institute of Health, Dental Materials and Biomaterial Research, 14197 Berlin, Germany; 2 Research Department, Sidra Medicine, Doha P.O. Box 26999, Qatar; cbollensdorff@sidra.org; 3 Pediatric Cancer Omics Lab., Cancer Group, Research Branch, Sidra Medicine, Doha P.O. Box 26999, Qatar; craynaud@sidra.org

**Keywords:** valvular interstitial cells, surface modification, vascular tissue engineering, hyaluronic acid, integrin, CD44, PTFE, PCL, bioconjugation

## Abstract

Tissue engineering concepts, which are concerned with the attachment and growth of specific cell types, frequently employ immobilized ligands that interact preferentially with cell types of interest. Creating multicellular grafts such as heart valves calls for scaffolds with spatial control over the different cells involved. Cardiac heart valves are mainly constituted out of two cell types, endothelial cells and valvular interstitial cells. To have control over where which cell type can be attracted would enable targeted cell settlement and growth contributing to the first step of an engineered construct. For endothelial cells, constituting the outer lining of the valve tissue, several specific peptide ligands have been described. Valvular interstitial cells, representing the bulk of the leaflet, have not been investigated in this regard. Two receptors, the integrin α9β1 and CD44, are known to be highly expressed on valvular interstitial cells. Here, we demonstrate that by covalently grafting the corresponding peptide and polysaccharide ligand onto an erodible, polycaprolactone (PCL), and a non-degradable, polytetrafluoroethylene (PTFE), polymer, surfaces were generated that strongly support valvular interstitial cell colonization with minimal endothelial cell and reduced platelet adhesion. The technology for covalent binding of corresponding ligands is a key element towards tissue engineered cardiac valves for in vitro applications, but also towards future in vivo application, especially in combination with degradable scaffold material.

## 1. Introduction

A wide range of materials are used for medical devices and other medical applications from a simple mechanical support up to the replacement of organ functions. The application of surface modification of biomaterials opens new possible applications that have been widely discussed [1]. It is important that the surface of the foreign material does not cause unwanted host responses that would impair the intended function. A desirable material would show both inert characteristics, such as antifouling [2,3,4], and promotion of colonization with specific cell types [5]. The methods most frequently used include plasma techniques [6] as well as chemical treatments, e.g., wet chemistry [7], to name but a few. Apart from simple coating with proteins of the extracellular matrix (ECM), more advanced techniques make use of covalent conjugation of ECM-derived recognition motifs such as the well-known “RGD” motif [5]. Additional peptides have been described addressing specific cell types, e.g., the amino acid sequences REDV and YIGSR were found to interact with endothelial cells in a selective manner [8,9,10]. Besides in vivo applications, the increasing interest in bioengineered scaffolds for organ replacement would benefit strongly from surface modifications targeting specific cell types to ensure a functional 3D reconstruction.

Valvular interstitial cells (VCs) are the predominant cell type of the heart valve, whereas endothelial cells (ECs) form the blood-contacting luminal layer [11]. To tissue engineer valves, surfaces that spatially favor either the adhesion and growth of VCs or ECs would be very beneficial [12]. An established strategy to engineer selective surfaces is to look for molecules that are highly—and possibly exclusively—expressed on the target cell. In the case of VCs, the receptors CD44 and the integrin α9β1 were found to match these criteria [13,14]. The ligand interacting with CD44 is known as the polysaccharide hyaluronic acid (HA). As an alternative approach, we employed a peptide derived from the protein tenascin C, which has been shown to bind specifically to α9β1.

Here, we report on the conjugation of a peptide and a polysaccharide ligand, specific for receptors highly expressed on VCs. Covalent grafting of the biomolecules is based on established methods for surface activation procedures combined with oriented peptide conjugation and a novel isocyanate-mediated HA grafting method.

Modifications were performed on non-degradable polytetrafluoroethylene as well as on a bio-erodible polycaprolactone and tested in cell culture to examine specific cell adhesion and platelet attachment.

## 2. Materials and Methods

Formamide, ethylene diamine (EDA), hexamethylene diisocyanate (HMDI), dimethylformamide, dimethyl sulfoxide, molecular sieve 3 Å, polycaprolactone (PCL, MW 70,000 g/mol), hyaluronic acid (HA), Calcein-AM, collagenase III, 3-(maleimido)propionic acid *N*-hydroxysuccinimide ester (NHS-Prop-Mal), and other standard chemicals were purchased from Sigma-Aldrich (St. Louis, MO, USA). Solvents were dried and stored over molecular sieve 3 Å. The PTFE sheet material was from Cadillac Plastic (Sulzbach, Germany). The peptide CPLAEIDGIELTY (95% purity) was custom synthesized by Activotec (Cambridge, UK). Human umbilical vein endothelial cells (HUVECs) and corresponding media were purchased from ThermoFisher Scientific (Waltham, MA, USA) (#C0035C).

### 2.1. Isolation of Sheep Valvular Interstitial Cells and Cell Culture

Sheep hearts (male and female) were obtained from a local abattoir in accordance with local (Qatar) regulations. Isolation was performed as previously described [15], with the following slight modifications. Briefly, valves were excised, first incubated in phosphate-buffered saline (PBS) containing antibiotics (5% penicillin/streptavidin) for 5 min, followed by the removal of the endothelial layer by treatment with collagenase III (Sigma, #C-0130, 0.6 mg/mL) in PBS for 20 min at 37 °C, followed by washing in sterile PBS buffer [16]. The remaining tissue was cut into pieces, placed into a flask with a small amount of medium (Dulbecco’s modified Eagle’s medium (DMEM), 10% fetal bovine serum (FBS), 1% penicillin/streptavidin), and left for adhesion for 1 h. Medium was then gently refilled to cover the tissue pieces, and cultivation was carried out at 37 °C, 5% CO_2_, and 95% humidity. After 3–4 days, outgrowing cells were harvested, split, and sub-cultured using standard procedures.

For further imaging, lentivirus was used to transduce cells with enhanced green fluorescence protein (eGFP) (LV-eGFP-0102, Cyagen, Santa Clara, CA, USA), according to the manufacturer’s recommendations. Briefly, cells were seeded in 24-well plates to be at 80% confluence on the day of transfection. Multiplicity of infection of 4 was used. Lentivirus and polybrene (final concentration of 10 μg/mL) were added to the cells and they were incubated for 24 h. Cells were subsequently cultured for 3 additional days. For homogenous eGFP expression, transduced cells were sorted on the basis of eGFP expression three consecutive times using Aria III cell sorter as previously published [17]. Briefly, after enzymatic dissociation using TrypLE™ enzyme, cells were stained for live/dead staining using a Zombie UV™ Fixable Viability Kit according to the manufacturer’s recommendation. Cells were rinsed with Dulbecco’s phosphate-buffered saline (DPBS) without Ca^2+^ and Mg^2+^. Cells resuspended in 1 mL of DPBS with 1 μL of Zombie UV™ were incubated 15 min at RT in the dark. Cells were then washed once with DPBS prior to filtration on 40 μm mesh. Single-cell suspension was analyzed by fluorescence-activated cell sorting (FACS) on a special order research products (SORP) FACSAriaIII (BD Biosciences, San Jose, CA, USA). Data were processed with FACSDiva 6.3 software (BD Biosciences). Doublets were excluded by FSC-W × FSC-H and SSC-W × SSC-H analysis. Dead cells were eliminated on the basis of UV fluorescence (DAPI channel); eGFP fluorescence was acquired with 488 nm blue laser excitation; and for emission, the FITC channel was used. The gating strategy is shown in Figure S1. Cells were sorted in a 15 mL tube containing 2 mL of fresh media using a 4 ways purity sort purity mask. Cells were then centrifuged and placed in culture in fresh media and allowed to grow for 2 weeks prior to a new sort. After two successive sorts, more than 98% of cells were found to be constitutively expressing eGFP (data not shown). Established eGFP-expressing cell lines were then used for further analysis.

HUVECs were grown in M199, complemented with 10% FBS and 1% penicillin/streptavidin. HUVECs were transduced similarly to VCs with eGFP for imaging purposes.

### 2.2. Sample Preparation and Surface Modification

**Figure 1:**
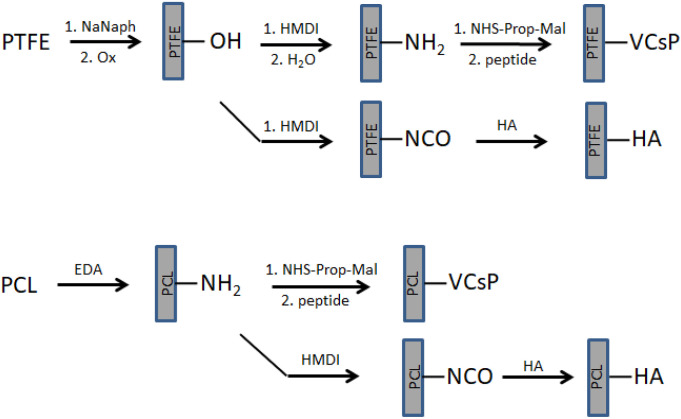
Schematic of surface modification of PTFE and PCL. After surface activation of the respective polymer, the peptide as well as HA were covalently immobilized in a multistep procedure. After treatment of PTFE in a combined elimination and oxidation reaction, the diisocyanate was used to couple the polysaccharide directly, and the peptide was immobilized after hydrolysis of the pendant isocyanate to a primary amine followed by grafting the peptide using a heterobifunctional crosslinker. The generation of free amino groups on PCL was accomplished by surface aminolysis using a diamine, and subsequent immobilization reactions were carried out in a similar way described for the modification of PTFE.

Schematic representations of the surface modification of PTFE and PLC are given in Figure 1.

Hydroxylated PTFE surfaces were prepared using a wet-chemical method that has been previously described [18]. In brief, PTFE was reacted with sodium naphthalenide and subsequently oxidized. Polymer discs (diameter 12 mm) bearing OH groups were treated with hexamethylenediamine (20% in dry hexane) for 2 h, rinsed copiously with hexane, and either hydrolyzed in water for at least 2 h (PTFE-NH_2_) or used directly (PTFE-NCO) in hyaluronan immobilization (see below).

For PCL, discs of 12 mm diameter and approximately 0.5 mm thickness were weighed and immersed in neat hexane. The swelling in hexane was monitored gravimetrically. After 24 h, the solvent was swabbed, and samples were weighed again. Aminated PCL material was prepared using ethylenediamine surface aminolysis, as detailed elsewhere [19].

Hexamethylenediamine in hexane was applied in a similar manner as described above, except that activated samples were immersed in neat dry hexane for 3 h before HA was conjugated.

HA was dissolved in dry formamide at 5 mg/mL following a published procedure [20] at 60 °C under mild shaking. The highly viscous clear solution, further diluted to 0.5 mg/mL in formamide, was used to incubate the isocyanate-activated PCL and PTFE samples for a minimum of 3 h or overnight. Polymer specimen (PTFE-HA, PCL-HA) were rinsed in formamide, then in hexane and water and dried. For peptide coupling, aminated polymer substrates were first reacted with 0.5 mg/mL NHS-Prop-Mal in 50% dimethylformamide/PBS for 2 h. We found that NHS-Prop-Mal is not soluble in 20% dimethylformamide as recommended by the supplier. After washing with buffer, the samples were treated with 0.25 mg/mL peptide solution in 50 mM carbonate buffer, pH 9, for at least 3 h. Unreacted peptide was removed by repeated washings with water. Samples were sterilized in 50% iso-propanol for a minimum of 30 min and rinsed in sterile PBS. HA-modified discs were pre-swollen in PBS prior to cell seeding.

### 2.3. Analytics

Static water contact angles were measured in triplicate on a Model G10 from Krüss (Hamburg, Germany) using a volume of 3 µL for each measurement. The presence of HA was qualitatively detected by incubating polymer samples with 1% Toluidine Blue in PBS for 5 min, followed by several rinses with water.

### 2.4. Cell Seeding and Platelet Adhesion Assay

Cells were seeded at 5 × 10^4^ in 2 mL of the respective medium onto each sample in 24-well plates. After 4 h, discs were gently rinsed with PBS, placed in a fresh plate, and refilled with 2 mL medium per well. Cell culture was performed at 37 °C, 5% CO_2_, and 95% humidity. Media was renewed every two days. After each incubation period, specimens were rinsed with PBS and immediately subjected to fluorescence microscopy using standard FITC filters.

Platelet-rich plasma was obtained from citrated full blood—following local legal regulations—by centrifugation for 20 min at 1500 rpm. The clear supernatant supplemented with 1 µL/mL Calcein-AM was used to incubate the samples for 45 min at 37 °C in the dark. Micrographs were obtained as mentioned above.

Pictures were taken at least in triplicates and quantitatively analyzed with ImageJ software (NIH, Bethesda, MD, USA). Quantification of cell adhesion was expressed as percentage coverage of total area.

## 3. Results

### 3.1. Efficient PTFE and PCL Modification

**Figure 2:**
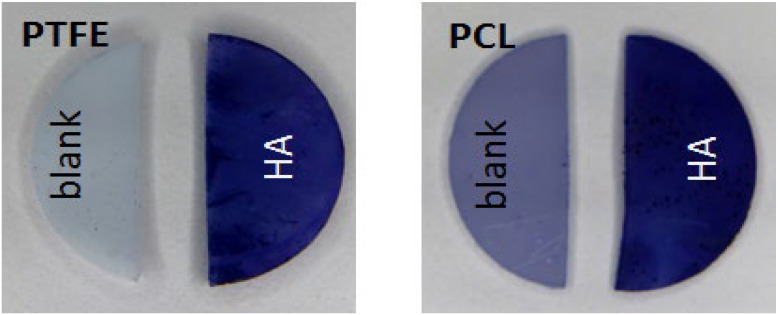
Qualitative detection of HA using Toluidine Blue staining. Whereas the dye caused only a slight background on both untreated materials, both PTFE and PCL modified with HA exhibited strong staining with Toluidine Blue, indicating the presence of HA.

In contrast to PTFE, which is completely insoluble in organic solvents, an appropriate solvent for HMDI had to be found that does not affect PCL to a significant degree. PCL swelling was therefore tested with hexane and found to be acceptable at 1.2 ± 0.12%. Neat HMDI was also checked as a swelling agent for PCL. Unexpectedly, about an 16% increase of the sample mass was observed. This plausibly explains the formation of a white precipitate (not shown) when HMDI-treated material was immersed in water after the chemical conjugation of HA, because the “leaking” of unreacted HMDI in contact with the aqueous environment leads to immediate polymerization of HMDI with its hydrolysis product. Pre-incubation with hexane prior to modification was able to prevent this unwanted phenomenon. In addition, FA was used as a solvent for the carbohydrate [20], and no interference with HMDI was observed. The presence of the acidic HA layer was visualized qualitatively using the cationic dye Toluidine Blue (Figure 2). Staining was visible on both polymers grafted with HA, but only a slight bluish background was found on untreated samples.

The untreated polymers displayed little, while the modified surfaces showed a strong blue staining, proving the presence of HA on the surface.

**Figure 3:**
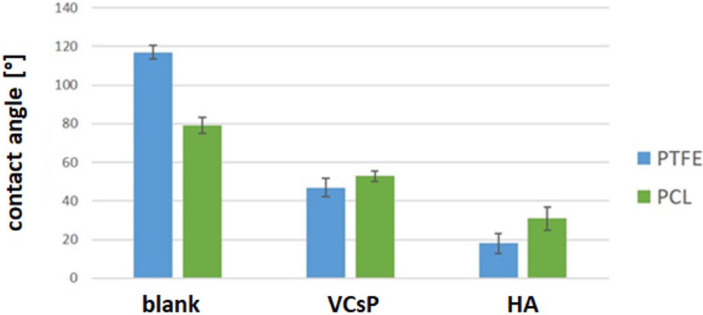
Contact angle measurements. Conjugation of hydrophilic biomolecules resulted in a significant improved wetting behavior on both polymers. This effect was more pronounced when immobilizing HA compared to the effect observed for the peptide VCsP.

As outlined in Figure 3, surface modification renders the highly (PTFE) as well as the moderately hydrophobic polymer (PCL) hydrophilic, as shown by the significant reduction in contact angles between pristine and modified polymers (Figure 3). The observed effect was more pronounced for HA than for the grafted peptide on both substrates.

Contact angles were measured for PTFE and PCL on pristine, HA- and VCsP-modified surfaces. Both modifications rendered the sample more hydrophilic. Error bars represent standard deviation of triplicate measurements.

### 3.2. Modification of PTFE and PCL Strongly Promoted VC but Not EC Adhesion and Reduced Platelet Attachment

**Figure 4:**
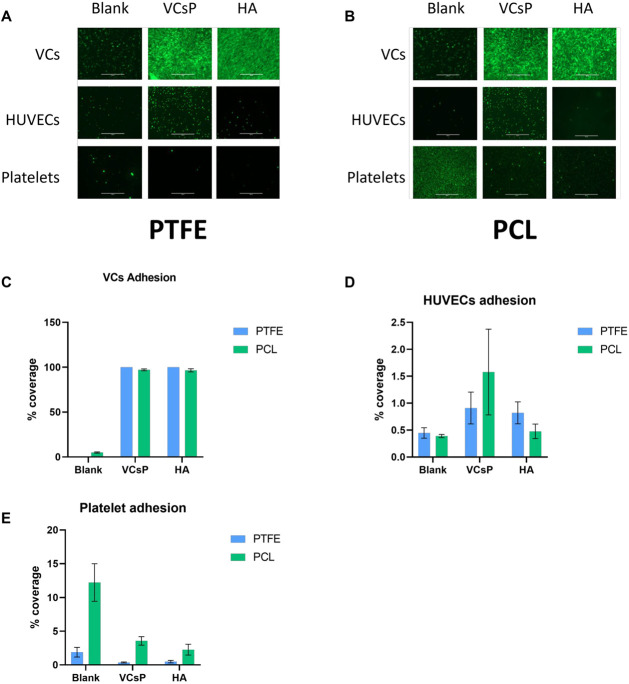
Cellular and platelet adhesion on pristine and modified material. (**A**). While pristine PTFE showed only poor adhesion of VCs, the conjugation of hyaluronic acid or VCsP allow VCs to colonize the substrate rapidly and efficiently. Confluence was reached after only one week. HUVEC adhesion was only marginally increased with HA or VCsP. Representative pictures of HUVEC and VC adhesion after one week are shown. Platelet adhesion was poor on pristine PTFE and almost completely abolished on modified material. Representative pictures of platelet adhesion after 45 min are shown. (**B**). Similar results were observed on PCL. Pristine material was a poor substrate for VC colonization, while immobilized HA or VCsP drastically increased the adhesion and growth of VCs. HUVEC adhesion was modestly increased in the presence of HA or VCsP. Representative pictures after one week of cell culture are shown. Platelets strongly attached to untreated PCL, which was drastically reduced in the presence of grafted HA or VCsP. (**C**). Quantification of cellular colonization by VCs on PTFE and PCL on untreated and modified polymers one week after seeding expressed as percentage coverage of total area. Pronounced growth was observed as a result of ligand immobilization. Error bars represent standard deviation of triplicate determinations using ImageJ. (**D**). Cellular colonization by HUVECs on untreated and modified PTFE and PCL after one week culture. Pristine and surface-grafted substrates did not support HUVEC growth to a large extent. (**E**). Quantification of platelet adhesion on pristine and modified PTFE and PCL. Each modification clearly reduced thrombogenic surface properties.

Cell culture was performed to assess each surface’s specificity for cell adhesion and thrombogenicity (Figure 4). Both untreated materials are poor substrates for cell adhesion and colonization (for VCs and HUVECs), even after one week of culture.

On the other hand, modification of PTFE resulted in confluency of VCs after only one week (*p* < 0.001 for both modifications). HUVEC adhesion was not promoted on these substrates (blank vs. HA: *p* = 0.046; blank vs. VCsP: *p* = 0.06). In addition, platelet attachment was reduced (blank vs. HA: *p* = 0.041; blank vs. VCsP: *p* = 0.029). Similar results were obtained for PCL as a substrate. VCs attached and grew to confluency in one week (*p* < 0.001 for both modifications). All three surfaces were unsuitable for HUVEC colonization as expected (blank vs. HA: *p* = 0.0.168; blank vs. VCsP: *p* = 0.039). Platelet attachment, not as pronounced as on untreated PTFE, was also highly reduced upon modification with both biomolecules (blank vs. HA: *p* = 0.002; blank vs. VCsP: *p* = 0.045).

## 4. Discussion

Tissue engineering of multi-cell-type organs requires spatially controlled colonization with appropriate cell types; in this context, ECs and VCs are the targeted cell types for a tissue-engineered heart valve.

We and others previously demonstrated that polymer surface modification with RGD (Arg-Gly-Asp peptide) or EC-specific peptides such as REDV (Arg-Glu-Asp-Val) and YIGSR (Tyr-Ile-Gly-Ser-Arg) [8,9,10] could promote EC colonization of the given polymers [18,19,21,22].

Aiming at adhesion of VCs, here we proposed a modification scheme making use of the immobilization of two ligands whose corresponding receptors (integrin α9β1 and CD 44) are highly expressed on VCs [13,14]. To the best of our knowledge, this strategy has not been pursued until now. As a rare example, Masters et al. [18] used fibronectin modification of a hydrogel to enhance VC proliferation. In addition, they found that RGD modification did not support VC colonization. Others observed VC growth on fibrous PCL scaffolds without any explicit modification, but this may most likely be attributed to the serum supplement that contains ECM components (e.g., fibronectin) that adsorbs unspecifically to most materials [23].

An immobilization strategy has to take into account the unique features of the respective scaffold material, such as solvent compatibility. The non-degradable PTFE—clinically used as expanded PTFE for vascular grafts—is of no concern in this respect because there is no known solvent. In contrast, PCL dissolves readily in many organic solvents (e.g., chloroform, dichloromethane, THF) at room temperature and in ethylacetate when slightly heated. We found hexane as an adequate solvent for surface modification of aminated PCL due to its ability to dissolve HMDI while not harming the polymer. Isocyanate conjugation was chosen because it may be assumed that a few OH groups of the polysaccharide molecule are consumed for coupling and the HA structure is not compromised to a large extent. Established methods for carbohydrate conjugation—notably, periodate oxidation—seem to be less suitable in this regard due to the partial oxidative ring opening of the sugar moieties [24]. Immobilization of HA via HMDI may therefore be regarded as beneficial for the preservation of the biological function of HA. We observed leaking of HMDI from PCL leading to a diffuse precipitate on the PCL samples. Simple soaking in hexane overnight was sufficient to prevent this polymerizate, which is probably caused by the reaction of partially hydrolyzed HMDI with the diisocyanate itself. Additionally, we found that the high swelling degree of PCL with HMDI was thus obviated (not shown). A putative reaction of HMDI with FA did not occur during the treatment time in agreement with earlier reports [25]. The use of organic solvents for the preparation of scaffolds poses a general safety concern. In comparison to chloroform, which is typically used for electrospinning of PCL without adverse effects [23], the toxicity of the two solvents used in this work (FA, hexane) are comparatively low [26]. Hexane is very volatile and can be expected to evaporate completely. The water-miscibility of FA enables extensive extraction from the sample material. Nevertheless, the coupling chemistry may be carried out with less toxic chemicals.

We demonstrated here that peptide as well as HA grafting impart hydrophilicity to the polymer surface. This result was expected since in both molecules, the peptide and the carbohydrate contain polar moieties, thus rendering the surface more wettable than the underlying base substrate. A high degree of hydrophilicity is not necessarily associated with robust cell adhesion, as can be seen from very hydrophilic surfaces, e.g., generated via PEGylation. Specific interactions between cell surface structures and a substrate are responsible for cellular attachment. This may be induced by non-specifically adsorbed ECM components of chemically grafted molecules.

The hydrophilic nature of modified surfaces probably contributes to the observed antifouling properties, i.e., reduced platelet adhesion, particularly regarding the lack of attachment sites (e.g., RGD motif in ECM proteins).

For obvious reasons, this effect was more pronounced on HA surfaces. Staining of acidic polysaccharides is readily feasible with the cationic dye Toluidine Blue, a qualitative proof of successful conjugation. The most important item in this work—the cell selectivity for VCs—was tested in cell culture. As initially supposed, VCs readily colonized peptide- as well as HA-modified substrates while HUVECs hardly attached. Given that VCs show a much higher growth rate compared to HUVECs, endothelialization is expected to be suppressed on modified surfaces. In addition, the non-thrombogenic properties created in this way might facilitate colonization and avoid the need for initial anticoagulation if blood contact is taking place. Due to the fast growth of VCs—in contrast to that of HUVECs—cell culture was only performed for one week until confluence was achieved. Co-culture of both cell types might be interesting in this regard.

Intended as a proof of principle for the successful preparation of VC specific surface modifications, several issues of VC biology were not addressed in this work, i.e., the undesirable differentiation of functional VCs into a smoother muscle cell phenotype, which may contribute to valve stiffening and calcification. Beside surface chemistry, the mechanical properties of the substrate or scaffold may also contribute to this unwanted outcome. In future studies, these points should be investigated in detail.

Taken together, appropriate surfaces were generated, specifically attracting VCs that may find application in valvular tissue engineering scaffolds, combining an outer layer attracting ECs with the inner bulk structure facilitating VC growth.

## 5. Conclusions

Covalent bioconjugation of appropriate valvular interstitial cell-selective ligands onto biomaterials produces surfaces that exhibit a pronounced preference for a cell type of interest. This approach might provide a novel route for heart valve tissue engineering.

## Data Availability

Data are available upon request from the corresponding author.

## Supplementary Materials

The following supporting information can be downloaded at: https://www.mdpi.com/article/10.3390/jfb13020070/s1, Figure S1: Gating hierarchy used for sort of eGFP modified VCs and HUVECs.
