# Supplementary material for: Surface Modification of Polytetrafluoroethylene and Polycaprolactone Promoting Cell-Selective Adhesion and Growth of Valvular Interstitial Cells
Source: J Funct Biomater. 2022 Jun 1;13(2):70. doi: 10.3390/jfb13020070 (PMC9225263; doi:10.3390/jfb13020070)

Supplemental information

Supplementary Figure S1. Gating hierarchy used for sort of eGFP modified VCs and ECs.

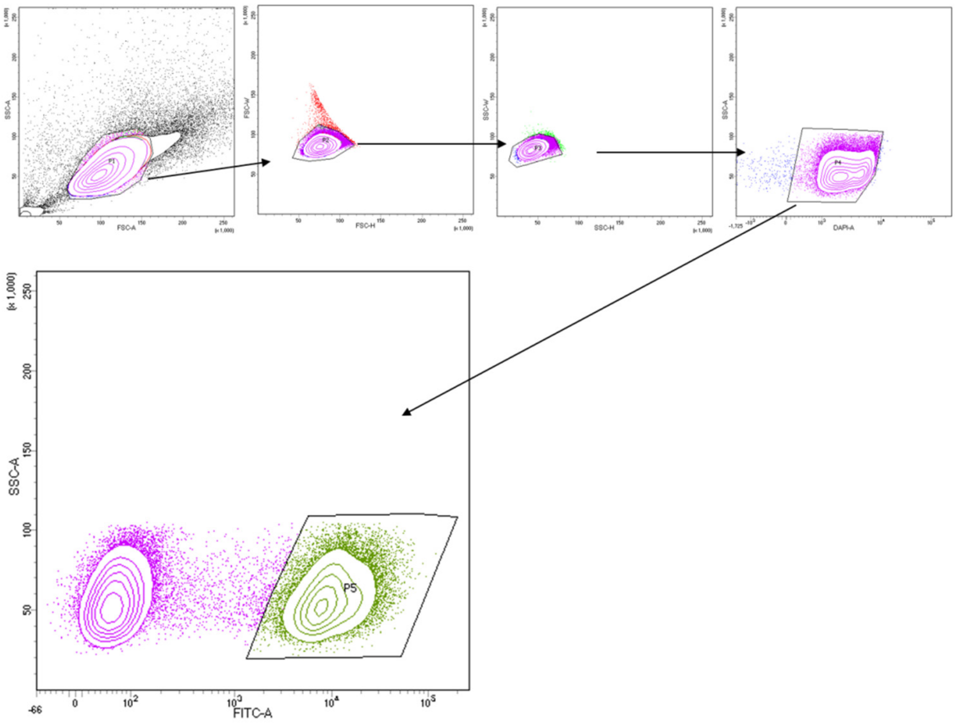

Supplement: Supplementary file 1 [file jfb-13-00070-s001.zip › jfb-1667045-supplementary.pdf]
